# Ouabain inhibition of Na/K-ATPase across the retina prevents signed refractive compensation to lens-induced defocus, but not default ocular growth in young chicks

**DOI:** 10.12688/f1000research.2-97.v1

**Published:** 2013-03-28

**Authors:** Melanie J Murphy, Sheila Gillard Crewther

**Affiliations:** 1 School of Psychological Science, La Trobe University, Melbourne, 3086, Australia

## Abstract

**Purpose:** The relevance of retinal integrity and energy pathways to ocular growth and induction of refractive errors has seldom been investigated. Thus, we used ouabain to target the channels that are essential for the maintenance of membrane potentials in cells, sodium potassium ATPase (Na/K-ATPase), to examine refractive compensation and ocular growth in response to lens-induced defocus in the chick.

**Methods:**  A single intravitreal injection of 1 mM ouabain in dimethyl sulfoxide (DMSO) carrier or DMSO alone was followed by monocular defocus with positive or negative 10 D lens (or no lens) from post-hatching days 5-9 under 12/12 hr light/dark conditions. Biometry and dark-adapted flash and electroretinography (ERG) were conducted on day 9, followed by immunohistological analyses.

**Results: **Ouabain inhibited differential ocular growth and refractive compensation to signed defocus compared to DMSO. By 4-days post-ouabain injection all components of the typical ERG responses to light had been eliminated, and widespread histological damage was apparent, though some ‘default state’ ocular growth was measurable. Immunohistochemistry demonstrated reduction in the specialized water channel Aquaporin 4 (AQP4) expression and increased evidence of caspase 3 expression (a cell death associated protein) in ouabain-treated eyes compared with DMSO alone.

**Conclusion:** The current study demonstrates that blockade of photoreceptor and inner retinal responses to light onset and offset by ouabain inhibits differential refractive compensation to optical blur, but does not prevent ocular growth.

## Introduction

Refractive compensation to optical blur in young animals has been attributed to environmentally-driven changes affecting the rate of ocular growth and axial dimensions of the eye ^1– 5^. To date only a few studies ^6– 8^ have investigated the impact of compromised retinal energetics and integrity on such growth, despite the retina being a highly metabolically active structure. Photoreceptor activity is highly dependent on aerobic glycolysis and the availability of adenosine triphosphate (ATP) energy resources ^9– 12^. In particular, an ATP source is needed to transport both the light induced changes in chloride, sodium and potassium ions out of the subretinal space (SRS) into the retinal pigment epithelium (RPE) and the obligatory accompanying fluid coming from the vitreous humour en route to the choroid. The sodium- [Na] potassium [K]-ATPase (Na/K-ATPase) channel is one of the most important channels involved in osmoregulation and fluid transport, particularly in the retina ^13^.

Na/K-ATPase activity is responsible for approximately 30 percent of total energy consumption in the body, and approximately 50 percent of the energy consumption in the retina ^14– 16^. Na/K-ATPase utilizes ATP hydrolysis to maintain electrolyte balance, and the associated electrochemical gradient across the cell membrane ^17, 18^ and cell volume by transporting Na ^+^ out of cells and K ^+^ in at a ratio of 3:2. Na/K-ATPase is critical to retinal function, regulating the Na ^+^ and K ^+^ gradients associated with the dark current in the photoreceptors, action potentials in the ganglion cells, Müller cell neurotransmitter uptake, light adaptation ^19^ and synaptic activity. In the light, the Na/K-ATPase maintains the high extracellular Na ^+^ concentration in the SRS, while also maintaining the potential difference across the apical membrane of the RPE and consequently driving the transport of ions and fluid from the retina across the RPE to the choroid ^20, 21^. Hence, changes in the pumping mechanism and/or expression of Na/K-ATPase by the selective inhibitor ouabain would be expected to alter the nature of phototransduction and affect outer and inner retinal neurotransmission.

Indeed, ouabain has been shown to affect the resting potential of the optic nerve, disrupting retinal metabolism and inducing spreading depression in neural tissue ^22^, and to affect cell density and the thickness of the inner plexiform layer (IPL) 2-days post-injection, with horizontal and amacrine cells being the primary sites of cell death. The extent of the morphological changes has been shown to affect the electrophysiological profile of the outer retinal response to a light stimulus in a dose-dependent manner ^15, 23^. At low concentrations, ouabain causes selective destruction of inner nuclear layer (INL) cells and the ganglion cell layer (GCL), while inducing reversible swelling in horizontal cells, preserving the Müller cells and photoreceptors ^24^. At higher concentrations, ouabain completely destroys the retina ^25, 26^.

To date the functional consequences of ouabain injection on ocular growth and refractive compensation to optical defocus have not been investigated in great depth. A recent investigation examining the effect of a relatively low dose of 1 mM ouabain on refractive compensation in chicks wearing positive lenses reported that application of the drug prevented the development of hyperopia ^27^. It was posited that this was due to the inhibition of Na/K-ATPase channels which then altered the nature of fluid transport across the RPE, leading to significant choroidal thinning and thus reduced refractive error. Unfortunately the effect of this dose of ouabain on photoreceptor function and general retinal morphology, choroidal dimensions, and on ocular growth and refractive compensation to negative lenses was not examined.

Thus, the current investigation aimed to examine the effect of a 1 mM dose of ouabain on refractive compensation and ocular elongation *per se* in response to negative as well as positive lenses or no optical adjustment. We hypothesize here that injection of ouabain, and subsequent inhibition of outer and inner retinal Na/K-ATPase activity, would not only prevent compensation to positive lenses as reported by Wong *et al.* ^27^, but also prevent abnormal ocular elongation, refractive compensation and myopia to negative lenses due to the inability of any retinal elements to detect defocus and elicit the required driving force needed to alter ocular volume. It is further hypothesized that there would be no systematic change in choroid thickness across the experimental lens groups due to the inhibition of retinal responses to light rather than as a result of active ocular volume reduction following ‘ouabain-driven change’ in fluid transport. Although ouabain would be expected to thin the choroid due to increased retinal adhesion ^28^, this effect is likely to be attenuated by the dilatory effect of dimethyl sulfoxide (DMSO) on this structure ^7^.

We also examined whether changes in refractive compensation and ocular growth were associated with altered outer retinal function (i.e. altered photoreceptor, bipolar, Müller and RPE cell function), as assessed by electroretinography (ERG), and retinal organization via histological and immunocytochemical analysis. Changes in the expression of the specialized glial water channel aquaporin 4 (AQP4) across the retina were measured in view of the previously reported association between ouabain and retinal adhesiveness and cellular edema ^28^. This work led us to expect altered intracellular and transretinal fluid production in negative lens versus no lens conditions. Apoptosis-related caspase 3 expression was also measured to assess the extent of cell death across the retina at the end of the chick rearing period.

## Methods

### Animals and rearing

All procedures were conducted in accordance with La Trobe University Animal Ethics Committee guidelines and adhere to the European Communities Council Directive of 24 November 1986 (86/609/EEC) and the ARVO Statement for the use of Animals in Ophthalmic and Vision Research. Well being of animals was monitored twice daily throughout the rearing period to ensure cleanliness and comfort. During surgical procedures, the level of anaesthesia was assessed continuously via toe squeeze, pupiliary reaction and respiration rate. Euthanasia for tissue collection was performed via decapitation while the animal was under heavy surgical anaesthesia.

A total of 71 male hatchling chicks (Leghorn/New Hampshire) obtained from a local commercial hatchery were raised in a light (12/12 hour day/night cycle) and temperature controlled (30 ± 0.5°C) enclosure. On post-hatching day 5 (P5), animals were randomly assigned to one of 3 lens conditions (±10 dioptre or no lens) and 2 drug conditions (ouabain [Sigma-Aldrich, MO, USA] in DMSO [Sigma-Aldrich, MO, USA] carrier or DMSO alone) and reared until post-hatching day P9. Ambient luminance was maintained constantly at 183 lux during the 12 hour day cycle via a 20W halogen light globe in the roof of the enclosure. The group sizes for each experimental condition are shown in Table 1.

**Table 1.** Mean (±SE) refractive state (S(RE)) and axial length (AL) for experimental eyes injected with ouabain or DMSO, and control eyes injected with DMSO.

		n	Experimental eye S(RE)	Control eye S(RE)	Experimental eye AL (mm)	Control eye AL (mm)
-10 D	Ouabain	10	-1.61 (±1.40)	1.15 (±0.37)	9.11 (±0.13)	8.83 (±0.07)
	DMSO	9	-8.90 (±0.46)	0.72 (±0.29)	9.18 (±0.13)	8.76 (±0.07)
‘No lens’	Ouabain	13	0.48 (±1.28)	0.69 (±0.50)	8.96 (±0.09)	8.86 (±0.05)
	DMSO	15	0.44 (±0.19)	0.33 (±0.18)	8.88 (±0.06)	8.79 (±0.06)
+10 D	Ouabain	9	-0.56 (±1.74)	1.39 (±0.50)	9.07 (±0.15)	8.84 (±0.06)
	DMSO	15	7.53 (±1.48)	0.70 (±0.29)	8.62 (±0.11)	8.77 (±0.05)

On day P5, chicks were anaesthetized (in the middle of the day cycle) with a intramuscular mixture of ketamine 45 mg/kg: xylazine 4.5 mg/kg and the right eye of each animal (experimental eye) was injected intravitreally with 5 μL of 1 mM ouabain in DMSO carrier solution (Sigma), to give an effective concentration of 2.5 μM in the eye assuming an ocular volume of 0.5 ml ^29^. This is a low-medium dose of ouabain selected for consistency of comparison with Wong *et al.* ^27^, and on the basis of previous reports of changes in retinal function and Na/K-ATPase activity in studies using effective concentrations of between 1.7 nM and 1 mM ^10, 30– 34^. The left eye of each animal was injected with the DMSO carrier as a within subject control. Monocular defocusing goggles (±10 D) customised from modified human polymethyl methacrylate (PMMA) contact lenses (8.1 mm in diameter) were then attached to Velcro© and affixed to the periocular feathers of the chicks for 4 days. A no lens control group was also used. The general health of the chicks and the cleanliness of lenses were monitored twice daily.

### Biometric analysis

On day P9, chicks were anaesthetized and both eyes were refracted by retinoscopy (Keeler, Vista Diagnostic Instruments, UK) and axial dimensions were obtained from the average of at least three A-Scan ultrasonography traces (A-Scan III, TSL: Teknar, Inc. St Louis, USA; 7 MHz probe).

### Electrophysiology

To ascertain the functional effects of ouabain on outer retinal integrity 4 days post-injection (P9), 4 additional ‘no lens’ animals that had been injected with ouabain in DMSO carrier at day P5 and reared for the same experimental period as the animals included in the biometric analyses were prepared for ERG recording and compared to 4 ‘no lens’ chicks of the same age, that had also been injected with DMSO intravitreally, and 3 that had been injected with phosphate buffered saline (PBS). A square wave with a 500 ms light on duration and offset at 500 ms stimulation protocol (150 mm Ganzfeld stimulator with peak luminance 50 cd/m ^2^, measured using a Tektronix J6523 narrow angle luminance probe [Tektronix, Inc, USA) was employed such that retinal ON and OFF responses could be separately observed. An intravitreal electrode (Ag/AgCl) was inserted under ketamine/xylazine anaesthesia (ketamine 45 mg/kg: xylazine 4.5 mg/kg i.m.) via a catheter placement unit, with scleral reference. Signals were recorded (under maintained anaesthesia) via a Powerlab amplifier (ADI, Australia) and band-pass filtered (0.3 – 1000 Hz). Twenty ERG recordings were obtained for each run, and 5 such runs were recorded consecutively for each animal. Grand mean averages with a 95% confidence interval for the mean recordings for each condition were determined using the IGOR common wave metrics software program (IGOR, Loswego, USA) for both drug conditions.

### Histological and immunohistochemical analysis

To assess the effect of ouabain on retinal morphology, patterns of cell death and the specialized AQP4 fluid transport mechanism, posterior eye cups of negative lens and ‘no lens’ animals were separated from the anterior portion of the eye at the midpoint of the ocular globe, and after the removal of the vitreous body, were then fixed in 4% paraformaldehyde (Sigma-Aldrich, MO, USA) for 30 minutes and washed in phosphate buffered saline (Sigma-Aldrich, MO, USA) three times before cryo-protection in 30% sucrose (Sigma-Aldrich, MO, USA) to inhibit crystal formation during freezing prior to embedding in Tissue-Tek OCT Compound (Sakura Finetek, USA) and cryosectioning in serial sections at 10 micron thickness. Samples were then stained with cresyl violet for general histological analysis or immunolabeled with AQP4 or caspase 3. Four slides, each with 3 sections, from 3 different animals per condition were examined under light microscopy using cresyl violet stain. The immunohistochemistry protocol and protein visualization was conducted as per the manufacturer’s instructions (Vector Laboratories Inc, CA, USA). This involved incubation of sections for 30 minutes in a 3% goat serum blocking solution (Vector Laboratories Inc, Burlingame, CA, USA) at room temperature, followed by primary antibody application overnight at 4°C with either rabbit anti-rat AQP4 antibody (1:20 dilution) or Caspase-3 (1:50; rabbit anti-rat; Millipore, CA, USA). Sections were then incubated for 30 minutes at room temperature with goat anti-rabbit biotinylated IgG (Vector Laboratories Inc, Burlingame, CA, USA) followed by incubation with ABC reagent (Vectastain ABC Kit with Rabbit IgG; Vector Laboratories Inc, CA, USA). Proteins were then visualised using a 3, 3'-diaminobenzidine (DAB) peroxidase substrate kit (DAB Peroxidase Substrate Kit, Vector Laboratories Inc, CA, USA). Slides were cover slipped with an anti-fade aqueous mounting medium (Gel Mount, Sigma-Aldrich, Australia). Ouabain- and DMSO-treated eyes were incubated together for each antibody localization protocol.

Protein localization and variation (channel and receptor expression) was examined via a light transmission microscope (Leitz Dialux 22 Microscope). Images were captured using Spot Flex Cooled CCD digital camera and Spot software (Diagnostic Instruments, Inc, MI, USA). The staining intensity of captured images was measured with *ImageJ* ( http://rsbweb.nih.gov/ij/; NIH, USA) analysis software.

### Data analysis

Biometric data in the preceding analyses utilised differences in values of the CE subtracted from the EE involved the following variables: refractive state, axial length, vitreous chamber depth and anterior chamber depth. The measurements for each animal were derived from an average of at least 3 scans, which were then averaged to obtain a mean difference for each dependant variable to control for within subject effects. Measurements were analysed via a series of 2 way Analysis of Variance (ANOVA) (3 lens × 2 drug) with an alpha = 0.05. Significant effects were further explored via Simple Main Effects Analysis, followed by either Student-Newman-Keuls (SNK) or Games-Howell (GH) Post Hoc testing when appropriate.

Histological analysis involved the examination of sections for evidence of any anatomical abnormalities. Layer thickness of the ganglion cell layer, inner retina (including inner and outer plexiform layers) and outer retina, including approximate transverse number and spatial distribution of cells per layer of retina was assessed. Higher magnification inspection was made of retinal neurons and photoreceptor outer segments and individual RPE cells to check for evidence of hyper- or hypo-osmotic cell profiles, nuclei with irregular profiles, or apparent holes or vacuoles that are indicative of cellular dehydration or edema, as these are the classically accepted signs of generalised toxicity and cell damage (see Liang *et al.* 1996 and Liang *et al.* 2004 ^35, 36^ for examples of these signs). Changes in the immunoexpression of AQP4 and caspase 3 were identified by alterations in the intensity of DAB staining across the retinal layers.

Grand mean average waves (with 95% confidence intervals) from the ERG recordings were obtained by averaging across the multiple recordings from the chicks in the DMSO and ouabain groups 4 days post injection. The main features of the ERGs analysed were the N50 ( *a*-wave - photoreceptor onset peak), the P105 ( *b*-wave bipolar amplitude peak), the response at light off (N500 - a surrogate measure of *c*-wave amplitude) and the P670 ( *d*-wave peak, the photoreceptor + Off Bipolar response to light offset).

## Results

**Figure 1.**
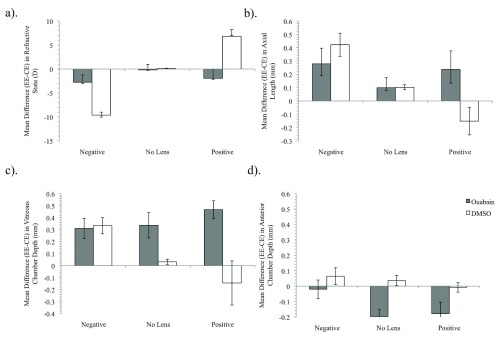
Biometric measures following single injection of ouabain or DMSO. Mean difference (± SE) measures of +/-10 D and ‘no lens’ eyes at 4 days for refractive state ( **a**), axial length (mm) ( **b**), vitreous chamber depth (mm) ( **c**), and anterior chamber depth (mm) ( **d**).

By day P9, 4 days after a single 5 μl injection of ouabain, it was clear that the treatment had notably altered the typical pattern of refractive compensation and ocular growth when compared with that seen in eyes with lens-induced defocus but injected with DMSO alone. Data presented in Table 1 and Figure 1a show that following ouabain injection, refractive compensation to both +10 D and -10 D defocus was severely inhibited, such that mean refractive state across all lens conditions varied by only 0.5 D while that of DMSO injected eyes varied by approximately 16 D.

Ouabain inhibition of Na/K-ATPase across the retina in chicks: biometric data Biometric measures for refractive state, axial length, vitreous chamber depth and anterior chamber depth.

The lack of directional refractive change observed in the ouabain condition is reflected in measures of axial length ( Figure 1b), with vitreous chamber depth and anterior chamber depth measures showing the same patterns of change ( Figures 1c and 1d, respectively).

Ouabain treatment led to a general increase in vitreous chamber depth and shallower anterior chamber depth, irrespective of the lens condition. In comparison, rearing following DMSO injection resulted in the typical sign-dependent changes of increased axial growth and deeper vitreous and anterior chamber depths in the -10 D lens condition with the opposite being found in the +10 D lens condition. Little change in biometric measures was observed in ‘no lens’ eyes for either intravitreal injection of ouabain or DMSO alone conditions, except for an increase in the anterior chamber depth of ouabain-injected eyes.

Two way (Drug × Defocus) ANOVA revealed significant interaction effects between the Drug and Defocus condition for measures of refractive state, axial length and vitreous chamber depth ( Table 2). The pattern of overall increased vitreous and overall decreased anterior chamber depths following single ouabain injection was supported statistically by a significant main effect for drug condition. Post hoc tests revealed significant differences between all lens conditions for refractive state, axial length and vitreous chamber depth in the DMSO condition, although the axial length and vitreous chamber depth for +10 D reared eyes were not significantly shorter than ‘no lens’ controls. Post hoc tests further confirmed that there was no significant difference between lens conditions for any measure for eyes injected with ouabain.

**Table 2.** ANOVA(+ power) results for comparisons between drug and lens conditions for measures of refractive state (RE), axial length (AL), vitreous chamber depth (VC) and anterior chamber depth (AC).

	RE	AL	VC	AC
Defocus	p < 0.001 (1-β = 0.999)	p = 0.02 (1-β = 0.747)	NS	NS
Drug	NS	NS	p < 0.001 (1-β = 0.989)	p < 0.001 (1-β = 0.989)
Defocus × Drug	p < 0.001 (1-β = 0.999)	p = 0.03 (1-β = 0.655)	p = 0.02 (1-β = 0.715)	NS

*NS, not significant.

## Histology and immunohistochemistry

**Figure 2.**
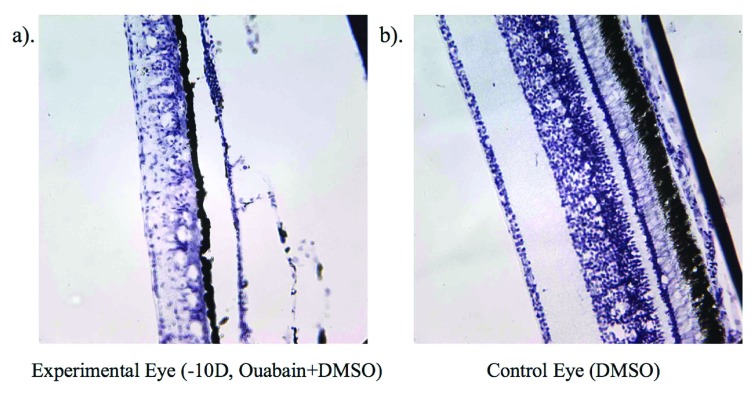
Cresyl violet-stained retina following single injection of ouabain in DMSO. Shows level of cell destruction on day 4 (P9) in chick wearing -10 D lens ( **a**) and a control eye ( **b**).

As shown in Figure 2, cresyl violet staining revealed widespread destruction of retinal elements in ouabain-injected eyes. Indeed, ouabain treatment induced fragility in tissue with all ouabain-injected eyes showing thinned choroids. DMSO alone did not induce any apparent histological damage and, as previously reported ^7^, induced a thicker choroid with dilated blood vessels as shown by cresyl staining. Of note, in the ouabain-treated eyes was the observation of cone degeneration, while rods and Müller cells were still present 4 days post-injection when compared with left eyes from the same animal treated with the DMSO carrier alone. Müller cell bodies appeared prominent and swollen, indicative of retinal edema particularly in -10 D lens condition eyes, consistent with the previous observations of Marmor ^28^ and Bringmann *et al.* ^37^. Significant disruption of the nerve fibre layer (NFL) was also apparent in the cresyl violet-stained retinae.

The expression of AQP4 in the NFL of ouabain-injected eyes was less than in the NFL of DMSO injected control eyes, with greater expression localised to the sublaminae A remnants of the IPL, particularly in the -10 D lens condition. The pattern of expression of caspase 3 staining was indicative of more widespread activation of apoptotic pathways across the retina following ouabain injection.

## Electrophysiology

**Figure 3.**
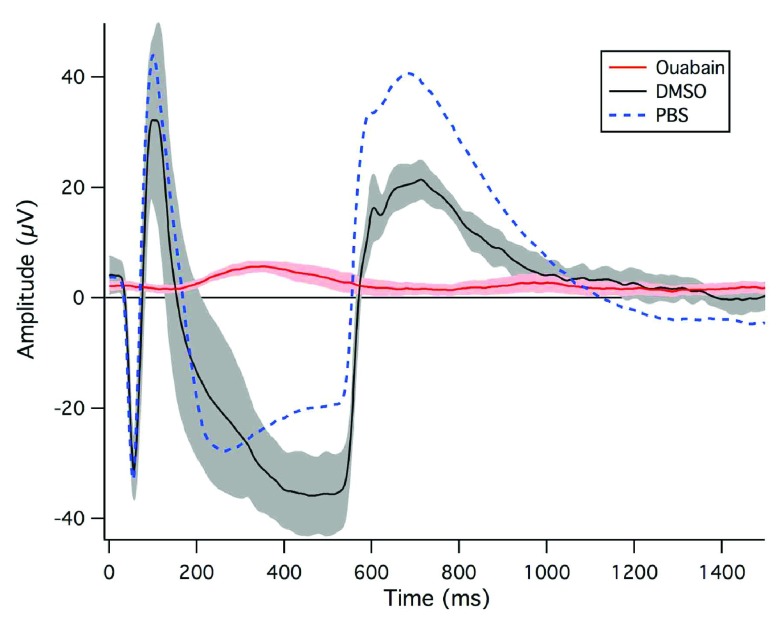
Electroretinography recordings from the eyes of chicks injected with ouabain, DMSO or PBS 4 days previously. Ouabain-injected chicks (n = 4) are shown by the red line, DMSO-injected chicks (n = 3) by the black line and PBS-injected chicks (n = 3) by the dotted blue line. Each recording is the average of 20 potentials over 5 runs per animal recorded during 500 ms light on followed by light offset. The 95% confidence intervals of the waves are indicated by the shaded regions surrounding the mean waves.

As can be seen in Figure 3, intravitreal injection of ouabain in DMSO but not DMSO alone affected the typical ERG response, in comparison to PBS-injected eyes. The normal ERGs following PBS injection obtained under our conditions of dark-adapted short flash response demonstrate a clear photoreceptor ( *a*-wave) and On bipolar (-wave) response occurring in conjunction with light onset, and an Off photoreceptor + OFF Bipolar response (referred to here as the -wave) being observed at light offset in ‘no lens’ eyes 4 days post-injection. As is obvious from Figure 3, and consistent with histological findings of photoreceptor and bipolar cell damage ( Figure 2), recordings 4 days after injection of ouabain-treated eyes did not show either photoreceptor responses to light onset or offset *a*-, *b*-, or *d* wave response to light onset or offset, in ‘no lens’ eyes. However, some evidence of a residual RPE ( *c*-wave) response was apparent in two of the four animals.

Ouabain inhibition of Na/K-ATPase across the retina in chicks: ERG data Electroretinography recordings from the eyes of chicks injected with ouabain, DMSO or PBS.

## Discussion

The electrophysiological results of the current study demonstrate that 1 mM of ouabain abolishes the photoreceptor ERG response to light onset and offset, and suppresses refractive compensation to both positive and negative lens-induced defocus but does not prevent ocular growth in ‘no lens’ conditions. In contrast, DMSO-injected eyes showed typical sign-dependent axial elongation or reduction in response to signed optical defocus ( Figure 2b). Biometric results also revealed shallower anterior chamber depths in ouabain-injected eyes. This is consistent with previous findings that ouabain acts on the ciliary body as well as the retina to inhibit the volume regulatory mechanisms in the epithelia in the anterior portion of the eye ^38, 39^.

The atypical ERG recordings obtained for ouabain-injected ‘no lens’ eyes are indicative of total suppression of photoreceptor and outer retinal function. In agreement with previous studies ^19^, our ERG recording data clearly demonstrated that the absence of a functional PR response following inhibition of the Na/K-ATPase transport system affects the regulation of electrical potential within the retina ^10, 23, 40– 42^ and leaves the eye unable to initiate any response to light onset or offset, and hence drive refractive compensation. The ERG recordings made in this study were also reminiscent of the effect of barium on the electrical response of the retina and RPE ^43– 46^. Barium, a potent K ^+^ channel inhibitor, has also been shown to eliminate signed refractive compensation and ocular growth to lens-induced defocus ^46^.

The biometric results suggest that the ouabain-induced loss of Na/K-ATPase activity in all cells in the retina and choroid eliminated the ability of the retina to detect blur and initiate sign-appropriate differential growth. Neither the biometric nor the immunohistochemical results indicate at what level in the retina this response was initiated. However, as indicated above, the ERG results show that functional activation of the retina from the level of the photoreceptors and beyond, has been eliminated. Indeed, the differential patterns of cell death across the retina are indicative of metabolic changes that would be expected following inhibition of Na/K-ATPase activity ^14, 47^. The swelling of the Müller cells observed in the current study is consistent with the changes observed in rat glioma cells undergoing osmotic challenge ^34, 48^. The widespread distribution of caspase 3 staining is further confirmation of the presence of apoptosis across the entire retinae of the ouabain-injected eyes.

Histological analysis also showed that the eyes injected with the carrier DMSO (that showed a typical refractive compensation response) all showed the expected characteristic choroidal thickening ^7^ irrespective of the lens group and resultant refraction. The choroidal response to DMSO confirmed our earlier observations ^7, 49^ of dissociation between the direction of ocular growth (and hence refractive compensation) and choroidal thickness. Light microscopy analysis revealed substantial disruption to retinal morphology and layer organization and no apparent systematic lens-induced change in choroidal thickness following ouabain injection.

Earlier studies have suggested that survival of the outer retina is necessary for refractive compensation to blur, though sign appropriate growth can occur following the inhibition of the inner retina ^7, 8, 50^. Indeed, the current findings are also consistent with reports of increased retinal adhesion due to swelling of photoreceptor outer segments and apical processes of the RPE ^28^. Additionally, the observation of choroidal thinning across all lens groups following injection of ouabain in DMSO carrier further demonstrates that changes in choroidal thickness do not dictate refractive compensation, as previously suggested ^7, 36, 49^.

Ouabain has been shown to destroy the ability of the retina to detect and respond to osmotic stress and adequately regulate cell volume ^28, 51^. This would be expected following ouabain-induced inhibition of Na/K-ATPase and the associated decrease in fluid flow out of retinal tissues and should be reflected by the expression of the retinal aquaporin AQP4 channel. Aquaporin channel expression has previously been shown to be involved in the mediation of fluid secretion and absorption in a variety of different functions within the eye ^52– 54^, including being associated with changes in ocular dimensions ^55, 56^. The growth changes seen in ouabain-treated animals in the current study are similar to the slower growth previously described in eyes compensating to positive lenses and the shift in the expression of AQP4 from primarily along the vitreal border of the NFL to the IPL layer in PBS- and DMSO-treated eyes to sublaminae A of the remaining IPL is also consistent with slowed vitreal volume change ^55^. These results further support a role for the participation of AQP4 expression on Müller cells in the regulation of retinal cell volume changes following alterations of ionic fluid balance in the eye and also highlight the importance of active, directional fluid and ion exchange across the retina in refractive development ^5, 57^.

Further, consistent with the observations of widespread caspase 3 labeling across the retina at 4 days post injection in the current study, Li *et al.* ^58^ demonstrated that the intensity of terminal deoxynucleotidyl transferase-mediated dUTP nick end-labeling (TUNEL) in rat retina increased over time from 12 hours post-ouabain injection, suggesting that ouabain initiated apoptotic processes within the retina. Micrographs from Li *et al.* ^58^ showed widespread disorganization of the retinal layers at all levels, with significant findings of destruction of the IPL. These histological characteristics bear marked similarities to the retinae assessed in the current study that exhibited vacuoles, swelling, altered Müller cell processes and overall decreased retinal thickness (mostly due to destruction of the inner retina).

Together, the current results show that 4 days after a single injection of ouabain, a substance that was expected to severely impair the energetic systems of all cells, the transepithelial potential had been abolished and fluid movement had been altered, apparently preventing the retina from detecting and responding to signed defocus. Importantly, recent evidence links Na/K-ATPase function with the effects of hypoxia ^10, 47^ and with changes in immune function in the retina ^31, 59– 61^, which could have important implications for conditions such as diabetic retinopathy ^62– 64^ and other metabolic conditions.

In conclusion, the results of the current investigation show that inhibition of Na/K-ATPase and photoreceptor responses to light onset and offset disrupt retinal organization and induce a non-sign-dependent choroidal thinning. This prevents signed ocular growth and refractive compensation to both positive and negative lens-induced defocus in the chick. Minimal non-sign-dependent growth of eyes still occurred irrespective of the functional state of the retina or the size of the choroid, also indicating that growth of the eye is a normal developmental condition even in the absence of a visual signal.
