# Supplementary material for: Ouabain inhibition of Na/K-ATPase across the retina prevents signed refractive compensation to lens-induced defocus, but not default ocular growth in young chicks
Source: F1000Res. 2013 Mar 28;2:97. [Version 1] doi: 10.12688/f1000research.2-97.v1 (PMC4257142; doi:10.12688/f1000research.2-97.v1)
Supplement: Peer review history

# Peer review: Referee response for version 1

**DOI:** 10.5256/f1000research.1001.r6307

**Published:** 2014-10-14

**Authors:** Shanta Sarfare

**Affiliations:** 1 Department of Ophthalmology, University of Florida, Gainesville, FL, USA

The paper is well written and the results are properly analyzed and interpreted.

An important control that has been performed but not described includes the PBS-injected eyes to demonstrate normal ocular growth at this age.

There is no figure showing the AQP4 immunohistochemistry or caspase labelling, when it is discussed extensively in the discussion section.

Figure 2 does not convincingly show increased swelling of Müller cells, and as such higher magnification images or specific antibody staining would be useful.

The ERG a-, b-, c- and d-waves are not labelled in figure 3. It would be difficult for the uninitiated to understand the figure without the labels.

The references should be thoroughly checked. For example, ref. 27 is incorrectly stated as published in IOVS, whereas it is actually only available as an ARVO abstract. 

It is indeed difficult to assess mechanism of oubain-induced refractive compensation due to the severe retinal toxicity of the chemical. Perhaps the authors ought to try lower doses with minimal toxic effects to tease out the true effect of ouabain?

I have read this submission. I believe that I have an appropriate level of expertise to confirm that it is of an acceptable scientific standard.

# Peer review: Referee response for version 1

**DOI:** 10.5256/f1000research.1001.r2232

**Published:** 2013-11-25

**Authors:** Debora Nickla

**Affiliations:** 1 Department of Biomedical Sciences and Disease, New England College of Optometry, Boston, MA, USA

This article describes the effects of a single injection of ouabain on ERGs, retinal histology, and the responses to positive and negative lenses in young chicks. Ouabain causes destruction of the retina as indicated by ERG amplitudes, caspase-3 expression and basic histology. It also prevents the compensation to both positive and negative lens-induced defocus. The writing is clear and concise, the results are convincing, and the science is sound. My only reservation is that it is unclear how much information about signal processing of defocus can be obtained by a drug that causes such massive damage to the retina, and so the results on the loss of the ability to compensate are not at all surprising.

In figure 1 the apparent lack of difference between control and experimental groups in the “no lens” axial length (figure 1b) seem to be due to the reduction in anterior chamber depth in the ouabain eyes (1d), because the vitreous chamber grows more than the DMSO controls (1c). It appears to me that ouabain increases vitreous chamber growth in these normal eyes. If this is so, then whether or not the drug affects “default” growth is not clear, because what is the default growth for these eyes? If the “default” is growing faster than normal, then it should be stated and clarified. For the same reason, the use of the term “default” in the title is ambiguous.

The authors state that "ouabain treatment led to a general increase in vitreous chamber depth and shallower anterior chamber depth, irrespective of the lens condition", but this is not so for the negative lenses when compared to DMSO controls (figs 1c and d). In the same paragraph, they say that ouabain increases anterior chamber depth in “no lens” eyes, but fig 1d shows a decrease in AC depth? This is confusing.

Because the choroids thinned in the drug-injected eyes in all groups does not indicate that “changes in choriodal thickness do not dictate refractive compensation”, as none of these eyes were compensating to the refractive error. If it is true that the drug-eyes were not growing faster than normal (and I’m not convinced that they were not, given figure 1c), then a more correct statement would be that choroidal thinning is not associated with faster-growing eyes.

I have read this submission. I believe that I have an appropriate level of expertise to confirm that it is of an acceptable scientific standard.
